# Hyper IgE Syndrome and Renal Cell Carcinoma

**DOI:** 10.1155/2017/7083451

**Published:** 2017-05-18

**Authors:** Neel H. Patel, Mark Ferretti, John L. Phillips

**Affiliations:** Department of Urology, New York Medical College, Valhalla, NY, USA

## Abstract

Hyper IgE Syndrome (HIES) is an immunodeficiency disorder characterized by increased serum levels of IgE, eczema, and recurrent cutaneous and pulmonary infections. In this report, we present, to our knowledge, the first documented case of renal cell carcinoma (RCC) found in a patient with HIES. The patient received infectious disease clearance prior to obtaining a partial nephrectomy which revealed clear cell histology. Both HIES and RCC have an immunological basis for their pathophysiology and may involve common pathways. Further studies may provide insight into any possible link and clinicians should be mindful of immunocompromised patients who present with risk factors for genitourinary malignancy.

## 1. Introduction

The Hyperimmunoglobulin E Syndromes (HIES) are rare primary immune deficiencies that are characterized by the triad of elevated serum IgE, rash, and recurrent bacterial infections of the skin and lung [1]. The incidence of HIES is expected to be 1 per 1,000,000. The disease was initially referred to as Job's syndrome, with reference to Biblical Job who was “smote with boils.” The autosomal dominant form is caused by mutations in the STAT3 gene which is a major signal transduction protein involved in wound healing, angiogenesis, immune pathways, and cancer [2]. It is characterized by nonimmunologic features such as skeletal, connective, and pulmonary abnormalities [1]. The autosomal recessive form is associated with viral and neurologic complications but otherwise is not well understood. Malignancies associated with Hyper Ig E Syndrome include Hodgkin's and Non-Hodgkin's lymphoma, as well as leukemia and those of the vulva, liver, and lung [3]. Here we present a case report of a patient with Hyper Ig E Syndrome and renal cell carcinoma. RCC represents a common and aggressive form of kidney cancer with approximately 64,000 new cases and 14,000 deaths per year within the United States [4]. This to our knowledge is the first case report showing manifestation of both conditions.

## 2. Case Report

**Figure 1:**
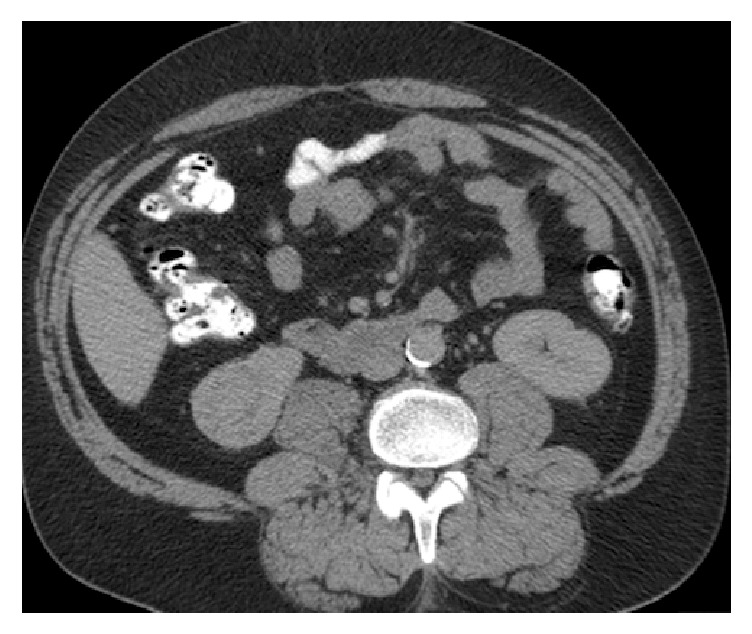
Axial image showing right kidney lower pole 4 cm renal mass with enhancement.

**Figure 2:**
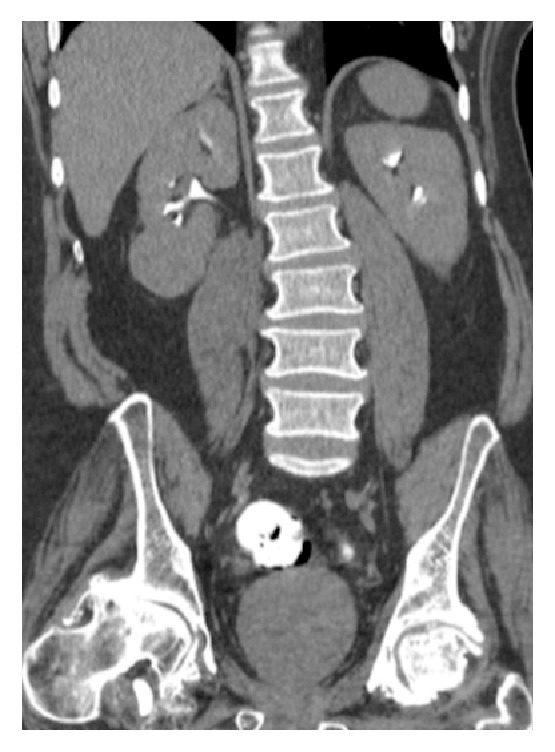
Coronal image of delayed phase demonstrating exophytic renal lesion within the right lower pole of the kidney.

A 56-year-old male was found to have a 4 cm exophytic right lower pole lesion upon work-up for complaints of back pain. The CT scan performed demonstrated enhancement (>20 HU) of the lesion and was suspicious for renal cell carcinoma as seen in Figures 1 and 2.

His comorbidities included a history of Autosomal Dominant Hyper IgE (Job's) Syndrome, sickle cell trait, alcoholic liver disease, avascular necrosis of the hip, polysubstance abuse, and depression. The patient described a history of recurrent skin infections with formation of multiple abscesses since he was a young child. These skin infections were managed both medically with the use of antibiotics and surgically with incision and drainage. He also had a history of respiratory infections consistent with his Hyper Ig E Syndrome. The patient continues to suffer from recurrent dental abscesses for which he requires tooth extractions and receives topical treatment for his HIES dermatitis. Serum IgE levels at the time of his malignancy work-up were found to be within normal limits. It had been over 10 years since the patient had engaged in any drug or alcohol abuse; however, given his alcoholic liver disease and HIES, he was at high risk for immunodeficiency. Appropriate work-up further revealed that the patient did not have human immunodeficiency virus and hepatitis B or C.

Treatment options were discussed with the patient including biopsy of the lesion, surgical intervention with partial/radical nephrectomy, and ablative procedures. The patient elected to undergo partial nephrectomy for treatment and diagnosis of the lesion. His preoperative work-up included medical as well as infectious disease clearance due to his immunocompromised state. Infectious disease consultation recommended standard preoperative surgical prophylaxis as the patient had no ongoing infection at the time of surgical planning.

Open partial nephrectomy was performed without any intraoperative complications and pathology of the specimen revealed pT1bNxMx clear cell renal carcinoma with Fuhrman Grade 2. The tumor was 4.1 cm in size and did not exhibit any sarcomatoid features and the tumor margins were negative.

## 3. Discussion

Hyper IgE Syndrome is associated with immunologic complications such as rash, boils, pneumonia, eczema, and lymphoma while nonimmunologic complications include characteristic facies, retained primary teeth, and joint hyperextensibility [1]. Malignancies such as lymphoma have been described, but there are no documented reports of an association with renal cell carcinoma. Renal cell carcinoma has been found to be associated with the immunodeficiency disorder chromosome 22q11 deletion syndrome [5].

Within the past two decades, immunotherapy has played a role in the treatment of renal cell carcinoma based on the infiltration of cancer tissue by tumor specific immune cells, specifically dendritic cells, T cells, natural killer cells, and macrophages [6, 7]. While T cells and natural killer cells play a role in the eradication of tumors, dendritic cells have the capacity to induce immunity or tolerance depending on their differentiation. Dendritic cells exhibit characteristics related to tumor immunoescape with high levels of matrix metalloproteinase-9 (MMP-9) and are known to promote tumor cell proliferation through promotion of TNF*α* and limited recruitment of T_H_1 polarized lymphocytes [8]. These immunological changes highlight the importance of immunotherapy as a key player in the management of metastatic renal cell carcinoma with various approaches such as cytokine-, antigen-, or dendritic cell-based immunotherapy [9].

With regard to HIES, the STAT3 gene when mutated affects signal transduction for many cytokines and growth factors that are responsible for cell survival, proliferation, migration, apoptosis, and inflammation in diverse cell types such as keratinocytes, hepatocytes, thymic epithelial cells, respiratory epithelial cells, neurons, lymphocytes, and macrophages [10]. It has also been demonstrated that impairment of interleukin 17 producing T helper cells (Th17) and induced regulatory T cells may account for the immunological abnormalities of HIES [11]. Mutations in the FOXP3 gene have also shown to play a role in immunodeficiency disorders.

In this report, we present a case describing the presentation of renal cell carcinoma in a patient with Hyper IgE Syndrome. Our patient presented with a long standing history of Hyper IgE Syndrome and its associated complications such as recurrent skin, dental, and pulmonary infections. Interestingly, during the time of our work-up his IgE levels were within normal limits which is a common finding in HIES patients as adults [12]. There is currently no evidence of a direct link between HIES and RCC, but immunological factors relevant to each condition may account for a possible association that has not yet been recognized. Recent studies have demonstrated an increase in infiltrating Th17 and FoxP3 cells within the tumor milieu of renal cell carcinoma [13, 14].

Given the relationship seen in both of these immunological disorders with a role being played by Th17 and FoxP3 it is possible that patients with these mutations and perhaps disorders may be at increased risk for development of renal cell carcinoma along with already well-established malignancies. Hypoxia Inducible Factor-1 (HIF-1) may also provide a key link between these two disorders. Studies have shown that HIF-1 regulated lysyl oxidase (LOX) is upregulated in vitro in cells infected with* Staphylococcus aureus* as well as in vivo in murine renal abscess and human skin abscess models [15]. Furthermore, LOX mRNA is increased in human clear cell renal cell carcinoma and is associated with decreased overall and metastasis-free survival [16].

Further research and studies need to be performed to delineate the immunological nature of malignant disease and any possible relationship with immunodeficiency disorders that has not yet been defined. Our findings present the need for careful consideration of RCC in immunodeficient patients presenting with risk factors, as well as the need for careful preoperative clearance and perioperative management to prevent unwarranted infectious complications.

## 4. Conclusion

Our findings demonstrate to our knowledge the first case presentation of renal cell carcinoma in a patient with Hyper Ig E Syndrome. Critical immunological factors play a role in the pathophysiology of each disease and may provide insight into a possible link between the disorders and further study is needed to delineate any shared immunological changes.
